# Inflammation-tumor burden interaction score stratifies survival after irinotecan-eluting bead chemoembolization for colorectal liver metastases

**DOI:** 10.3389/fmed.2026.1751660

**Published:** 2026-03-12

**Authors:** Tolga Doğan, Semra Taş, Emre Hafızoğlu, Taliha Güçlü Kantar, Burçin Çakan Demirel, Emre İspir, Erdem Çomut, Muhammet Arslan, Burcu Yapar Taşköylü, Atike Gökçen Demiray, Arzu Yaren, Gamze Gököz Doğu

**Affiliations:** 1 Department of Medical Oncology, Denizli State Hospital, Denizli, Türkiye; 2 Department of Medical Oncology, Pamukkale University Faculty of Medicine, Denizli, Türkiye; 3 Department of Medical Oncology, Afyonkarahisar State Hospital, Afyonkarahisar, Türkiye; 4 Department of Medical Oncology, Istanbul Medipol University, Istanbul, Türkiye; 5 Department of Biochemistry, Izmir City Hospital, Izmir, Türkiye; 6 Department of Pathology, Pamukkale University Faculty of Medicine, Denizli, Türkiye; 7 Department of Radiology, Pamukkale University Faculty of Medicine, Denizli, Türkiye

**Keywords:** colorectal liver metastases, irinotecan-eluting bead chemoembolization, prognostic score, risk stratification, systemic inflammation, tumor burden

## Abstract

**Background:**

Transarterial chemoembolization with irinotecan-loaded drug-eluting beads is an established locoregional option for selected patients with colorectal liver metastases who are not candidates for resection or ablation, but survival outcomes remain heterogeneous and simple prognostic tools are lacking.

**Methods:**

In this retrospective single-center study, we analyzed 70 patients treated between 2015 and 2024 to investigate whether the interaction between systemic inflammation and liver tumor burden can stratify survival after this procedure. Dynamic inflammatory change was quantified using the difference in the C-reactive protein-to-albumin ratio (ΔCAR) between baseline and early post-treatment assessments, and liver tumor burden was categorized by the number of metastases (< 5 vs. ≥ 5). These components were integrated into a composite chemoembolization–tumor burden–inflammation balance score (CT-IBS), and its association with early radiologic response, progression-free survival, and overall survival was evaluated using Kaplan–Meier analysis, receiver operating characteristic curves, and multivariable Cox regression.

**Results:**

At a median follow-up of 20.3 months, median progression-free and overall survival were 9.1 and 18.9 months, respectively, and early radiologic response (complete or partial) was observed in 75.7% of patients. Higher ΔCAR and a greater number of liver metastases were independently associated with inferior overall survival. The CT-IBS stratified patients into three distinct prognostic groups (median overall survival 27.3 vs. 17.8 vs. 8.6 months; *p* < 0.001; area under the curve 0.703).

**Conclusion:**

Integrating dynamic inflammatory changes with liver tumor burden yields a simple, reproducible classification that may support risk stratification, patient selection, and post-treatment surveillance after irinotecan-eluting bead chemoembolization for colorectal liver metastases.

## 1 Introduction

Colorectal cancer (CRC) remains a major global health burden, with nearly 1.9 million new cases and approximately one million deaths reported each year (1). The liver is the predominant site of metastatic spread, and up to one-third of patients present with or subsequently develop colorectal liver metastases (CRLM) during the course of their disease (2, 3). Although hepatic resection is the only potentially curative option, most patients are not candidates because of extensive tumor burden, anatomically unresectable disease, comorbidities, or extrahepatic involvement (3, 4). Despite advances in systemic chemotherapy and targeted therapies, outcomes for patients with unresectable, liver-dominant metastases remain suboptimal, underscoring the need for effective liver-directed modalities within multidisciplinary treatment algorithms (5, 6). Locoregional transarterial approaches, including hepatic arterial infusion chemotherapy and transarterial chemoembolization (including drug-eluting bead techniques), have increasingly been explored in patients with inoperable colorectal cancer and colorectal liver metastases; however, indications, drug regimens, and combination strategies remain heterogeneous, and no consensus has yet been established (7).

For patients who are ineligible for resection or thermal ablation, liver-directed locoregional therapies are central components of multimodal management. Irinotecan-based transarterial chemoembolization with drug-eluting beads (DEBIRI-TACE) allows high intratumoral drug delivery while inducing selective arterial ischemia. Long-term data from the 26-year Frankfurt program have shown that transarterial approaches, alone or in combination with ablative procedures, can achieve meaningful clinical outcomes even in heavily pretreated CRLM populations when used within structured multidisciplinary strategies (8). Contemporary DEBIRI- focused reviews similarly indicate that irinotecan-loaded drug-eluting microsphere TACE is feasible, well tolerated, and capable of providing durable hepatic disease control in unresectable or treatment-refractory CRLM (9). In parallel, the prospective CIREL cohort demonstrated clinically relevant survival with irinotecan-based TACE and identified Eastern Cooperative Oncology Group performance status, hepatic tumor burden, lesion size, and prior systemic therapy as independent predictors of outcome, underscoring the importance of careful patient selection (6).

Nevertheless, despite accumulating clinical experience, there is still no simple, unified framework to estimate prognosis or guide routine patient selection for irinotecan-based TACE in unresectable CRLM (6–9). In particular, the combined effect of systemic inflammatory response and liver tumor burden after DEBIRI-TACE has not been operationalized into a practical prognostic tool for everyday use. This gap emphasizes the need for pragmatic analyses that use readily available clinical and imaging parameters to define prognostic subgroups. It should be noted that in the present study DEBIRI-TACE was mainly used for patients considered unsuitable for curative liver resection at the time of multidisciplinary evaluation rather than as a predefined downstaging strategy. In this context, we investigated whether integrating dynamic inflammatory changes with hepatic tumor burden could stratify survival after DEBIRI-TACE in unresectable CRLM and developed a simple inflammation–tumor burden interaction score to support risk-adapted decision-making.

## 2 Materials and methods

### 2.1 Study design and patients

This retrospective cohort included consecutive patients with CRLM who underwent transarterial chemoembolization (TACE) with irinotecan-loaded drug-eluting beads between January 2015 and December 2024 at a tertiary oncology center. Clinical, laboratory, imaging, treatment, and survival data were obtained from electronic medical records. The study was approved by the Pamukkale University Non-Interventional Clinical Research Ethics Committee (Approval No: E- 60116787-020-767194; 16 October 2025) and conducted in accordance with the Declaration of Helsinki.

**FIGURE 1:**
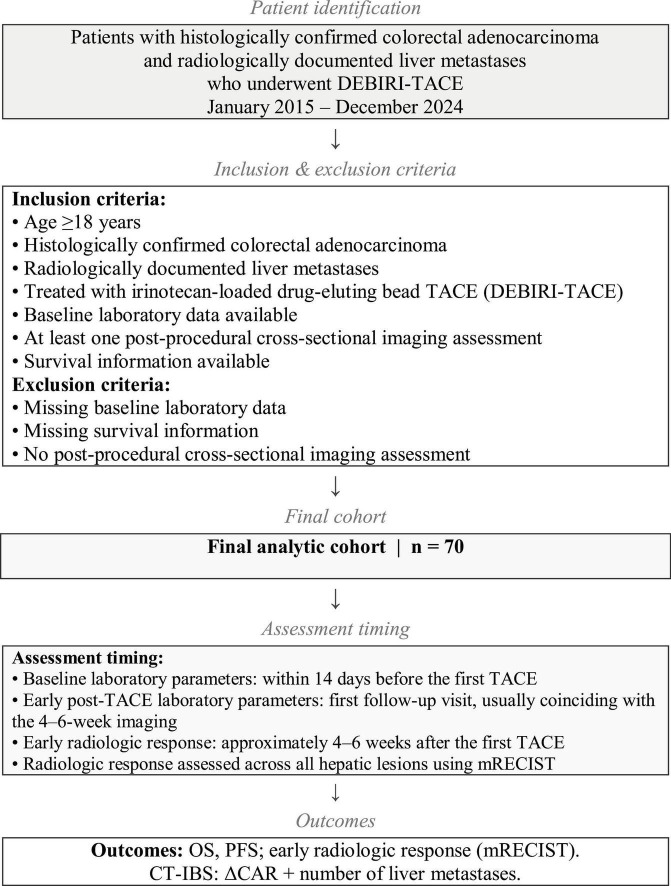
Flowchart illustrating patient selection and study process. All radiologic assessments were performed using cross-sectional CT/MRI and evaluated across all hepatic lesions by mRECIST. DEBIRI-TACE, transarterial chemoembolization with irinotecan-loaded drug-eluting beads; ΔCAR, change in the C-reactive protein-to-albumin ratio from baseline to early post-treatment assessment; CT-IBS, chemoembolization–tumor burden–inflammation balance score; mRECIST, modified Response Evaluation Criteria in Solid Tumors; OS, overall survival; PFS, progression-free survival.

Eligible patients were ≥ 18 years old, had histologically confirmed colorectal adenocarcinoma and radiologically documented liver metastases, and received irinotecan-loaded drug-eluting bead TACE. Patients without baseline laboratory data, survival information, or at least one postprocedural cross-sectional imaging assessment were excluded. All patients were discussed at a multidisciplinary tumor board including hepatobiliary surgery, medical oncology, and interventional radiology prior to treatment. The decision to proceed with DEBIRI-TACE rather than surgical resection was based on anatomical unresectability, insufficient hepatic reserve, extrahepatic disease burden, patient comorbidities, or patient preference, as determined on an individual basis. Resection of the primary colorectal tumor was not required for study inclusion. Patients with an intact primary tumor were eligible provided the primary lesion was asymptomatic and liver-directed therapy was considered the immediate treatment priority following multidisciplinary tumor board evaluation. Primary tumor resection status was recorded as a baseline characteristic and included as a covariate in survival analyses. Previous systemic therapy was not an exclusion criterion. Patients could have received systemic treatment before DEBIRI-TACE according to prior treatment history, and additional systemic therapy after the procedure was administered based on multidisciplinary clinical evaluation. Seventy patients met all criteria and were included in the final analysis. A flowchart summarizing patient selection and the study process is provided in Figure 1.

### 2.2 TACE procedure

Irinotecan-loaded drug-eluting beads (DC Bead^®^, Biocompatibles/BTG International, Farnham, Surrey, United Kingdom; currently Boston Scientific) measuring 100–300 μm were used throughout the study period. Each vial was loaded with 100 mg irinotecan according to the institutional protocol. Embolization was performed selectively through the hepatic arterial branches supplying the tumor-bearing segments until near stasis of arterial flow was achieved. All procedures were carried out by the same interventional radiology team using a standardized technique. In patients with multiple liver metastases, embolization was performed superselectively through the hepatic arterial branches supplying all angiographically visible lesions rather than targeting a single index lesion. Radiologic response was assessed across all hepatic lesions using mRECIST criteria. Contrast-enhanced computed tomography (CT) or magnetic resonance imaging (MRI) was performed approximately 4–6 weeks after the first TACE session to assess early radiologic response. The indication for a second TACE session was discussed in a multidisciplinary tumor board, based on radiologic response, liver function, and clinical status. Five patients (7%) received more than one TACE session, and none received more than two. Overall survival (OS) and progression-free survival (PFS) were calculated from the date of the first TACE; additional sessions did not reset survival time.

### 2.3 Clinical, laboratory, and imaging variables

Baseline clinical variables included age, sex, Eastern Cooperative Oncology Group performance status (ECOG PS), primary tumor location, resection of the primary tumor, presence of extrahepatic disease, prior lines of systemic therapy, and post-TACE systemic treatments. Molecular characteristics comprised KRAS, BRAF, and HER2 status, and microsatellite instability (MSI) where available.

Baseline laboratory values were obtained within 14 days before the first TACE session and included complete blood count, liver function tests, bilirubin, albumin, lactate dehydrogenase (LDH), and C-reactive protein (CRP). Early post-TACE laboratory values were recorded at the first follow-up visit, usually coinciding with the 4–6-week imaging.

Tumor burden was characterized by the number and maximum diameter of liver metastases on baseline imaging. For primary analyses, hepatic tumor burden was categorized as < 5 versus ≥ 5 liver metastases. Early radiologic response at approximately 1 month was abstracted from CT or MRI reports and classified as complete response, partial response, stable disease, or progression; in secondary analyses, complete or partial response was grouped as early response.

### 2.4 Inflammatory indices and CT-IBS

Systemic inflammatory indices were calculated from routine blood parameters. The derived neutrophil-to-lymphocyte ratio (dNLR) was defined according to previously published descriptions using the absolute neutrophil count and total white blood cell count. The C-reactive protein-to-albumin ratio (CAR) was obtained by dividing serum CRP (mg/L) by serum albumin (g/L). Baseline dNLR was categorized using the established cutoff of 3, and baseline CAR was dichotomized at the cohort median.

Dynamic inflammatory change was assessed using the difference between post-treatment and baseline CAR values (ΔCAR). ΔCAR was dichotomized at the median value.

To capture the interaction between systemic inflammation and hepatic tumor burden, we constructed the chemoembolization–tumor burden–inflammation balance score (CT-IBS). Two binary components were used: ΔCAR (low vs. high, based on the median) and liver tumor burden (< 5 vs. ≥ 5 metastases). Each adverse factor (high ΔCAR and ≥ 5 liver metastases) was assigned one point, yielding three CT-IBS categories: CT-IBS 0 (no adverse factors), CT-IBS 1 (one adverse factor), and CT-IBS 2 (both adverse factors). CT-IBS was the main exposure variable for survival analyses.

### 2.5 Survival outcomes

OS was defined as the time from the first TACE session to death from any cause; patients alive at last contact were censored at the date of last follow-up. PFS was defined as the time from the first TACE to the first documented radiologic progression or death; patients without an event were censored at the date of their last radiologic evaluation.

### 2.6 Statistical analysis

Continuous variables were summarized as medians with interquartile ranges (IQRs), and categorical variables as counts and percentages. Group comparisons were performed using the Mann–Whitney U or Kruskal–Wallis tests for continuous variables and the chi-square test or Fisher’s exact test for categorical variables, as appropriate.

OS and PFS were estimated using the Kaplan–Meier method, and survival curves were compared with the log-rank test. Univariable Cox proportional hazards models were used to explore associations between candidate prognostic factors and OS or PFS. Variables with *p* < 0.10 in univariable analyses, together with clinically relevant covariates (age, ECOG PS, extrahepatic disease, number and size of liver metastases, prior systemic therapy, post-TACE systemic therapy, and inflammatory indices including dNLR, CAR, CRP, ΔCAR, and CT-IBS), were considered for multivariable Cox models. To limit overfitting, the number of covariates in each model was restricted in relation to the number of observed events, and clearly overlapping variables (for example, different measures of tumor burden) were not entered simultaneously.

The discriminative ability of CT-IBS and selected inflammatory markers for OS and PFS was explored using receiver operating characteristic (ROC) curve analysis, and the area under the curve (AUC) was calculated. These analyses were descriptive and intended to complement the Cox models.

All statistical tests were two-sided, and *p* < 0.05 was considered statistically significant. Patients with missing key baseline or outcome data were excluded by design; all analyses were performed on complete cases using IBM SPSS Statistics, version 26 (IBM Corp., Armonk, NY, United States).

## 3 Results

Seventy patients with colorectal liver metastases who underwent TACE were included. The median age was 62 years (range, 33–80), and 57.1% were male. Most had an ECOG performance status of 0–1 (94.3%), and 81.4% had previously undergone resection of the primary tumor. Primary tumors most often arose from the left colon, followed by the rectum and right colon, and all were adenocarcinomas. Molecular profiling showed RAS (KRAS/NRAS) mutations in 40.0% of patients and BRAF mutations in 2.9%, with no cases of HER2 amplification; 45.7% had RAS/BRAF wild-type disease, while molecular data were unavailable in 10.0% of the cohort. Baseline clinical and tumor characteristics are presented in Table 1.

**TABLE 1:** Baseline demographic and tumor characteristics of patients undergoing TACE (*n* = 70).

Variable	n (%) or median (range)
Age (years)	62 (33–80)
**Sex**	
Male	40 (57.1)
Female	30 (42.9)
**Performance status (ECOG)**	
0–1	66 (94.3)
≥ 2	4 (5.7)
Primary tumor resection	57 (81.4)
**Primary tumor site**	
Right colon	12 (17.1)
Left colon	40 (57.1)
Rectum	18 (25.7)
Histology	Adenocarcinoma (100)
**Smoking history**	
Ever-smoker	26 (37.1)
Never-smoker	44 (62.9)
**Molecular profile**	
RAS mutation (KRAS/NRAS)	28 (40.0)
BRAF mutation	2 (2.9)
HER2 amplification	0 (0)
RAS/BRAF wild-type	32 (45.7)
Unknown	7 (10.0)

TACE, transarterial chemoembolization; ECOG, Eastern Cooperative Oncology Group; RAS, rat sarcoma viral oncogene (KRAS/NRAS); BRAF, v-raf murine sarcoma viral oncogene homolog B; HER2, human epidermal growth factor receptor 2.

Most patients (67.1%) had received at least one line of systemic therapy before TACE, predominantly FOLFOX- or FOLFIRI-based regimens, often in combination with bevacizumab. After TACE, 48.6% received additional systemic treatment, again mainly fluoropyrimidine– oxaliplatin or fluoropyrimidine–irinotecan combinations, including XELOX plus bevacizumab in a subset. Overall, bevacizumab-containing regimens were used in 37.1% of patients following the procedure, and 11.4% received anti-EGFR–based treatments.

Early radiologic assessment at approximately 1 month showed complete response in 30.0%, partial response in 45.7%, and progressive disease in 24.3% of patients according to mRECIST. The median number of liver metastases was three, and the median diameter of the largest lesion was 38 mm. Treatment characteristics and early radiologic outcomes are summarized in Table 2. Molecular subtypes (KRAS, BRAF, and HER2) were not associated with early radiologic response (*p* = 0.996).

**TABLE 2:** Treatment characteristics and early radiologic response (*n* = 70).

Variable	n (%) or median (range)
Prior systemic therapy	47 (67.1)
FOLFOX	19 (27.1)
FOLFIRI + bevacizumab	15 (21.4)
FOLFOX + bevacizumab	11 (15.7)
Other regimens (XELOX, capecitabine, etc.)	2 (2.9)
Systemic therapy after TACE	34 (48.6)
FOLFOX	12 (17.1)
XELOX	9 (12.9)
FOLFIRI	6 (8.6)
Other regimens	7 (10.0)
**Targeted therapy after TACE**	
Bevacizumab-based	26 (37.1)
Anti-EGFR-based	8 (11.4)
Primary tumor resection	57 (81.4)
Number of liver metastases	3 (1–9)
Largest lesion diameter (mm)	38 (12–110)
**Radiologic response at 1 month (mRECIST)**	
Complete response	21 (30.0)
Partial response	32 (45.7)
Progressive disease	17 (24.3)

TACE, transarterial chemoembolization; FOLFOX, 5-fluorouracil/leucovorin/oxaliplatin; FOLFIRI, 5-fluorouracil/leucovorin/irinotecan; XELOX, capecitabine/oxaliplatin; EGFR, epidermal growth factor receptor. Targeted therapies were administered in combination with chemotherapy backbones; therefore, these categories are not mutually exclusive. Percentages are calculated based on the total study population (*n* = 70).

At a median follow-up of 20.3 months, median PFS was 9.1 months (95% CI, 7.2–11.4), and median OS was 18.9 months (95% CI, 15.3–22.5). The 1-year PFS and OS rates were 34.7 and 71.6%, respectively. Survival time was calculated from the date of the first TACE procedure.

In the multivariable Cox model for OS, two variables remained independently prognostic. A higher baseline CAR was associated with shorter survival (HR 1.32, 95% CI 1.07–1.64; *p* = 0.010), while a lower dNLR (below 3) predicted more favorable outcomes (HR 0.39, 95% CI 0.21–0.71; *p* = 0.002). Incorporating the number of hepatic metastases into the model did not alter these associations. Although ΔCAR did not retain statistical significance after adjustment, patients with higher ΔCAR consistently showed shorter survival in univariable analyses, indicating that dynamic change in CAR after TACE carried additional prognostic information.

In the multivariable model for PFS, having five or more hepatic metastases was independently associated with an increased risk of progression (HR 5.33, 95% CI 1.13–25.14; *p* = 0.035). When the number of metastases was excluded from the model, the post-TACE CRP-to-albumin ratio remained an independent predictor of earlier progression (HR 1.04, 95% CI 1.01–1.07; *p* = 0.005).

**FIGURE 2:**
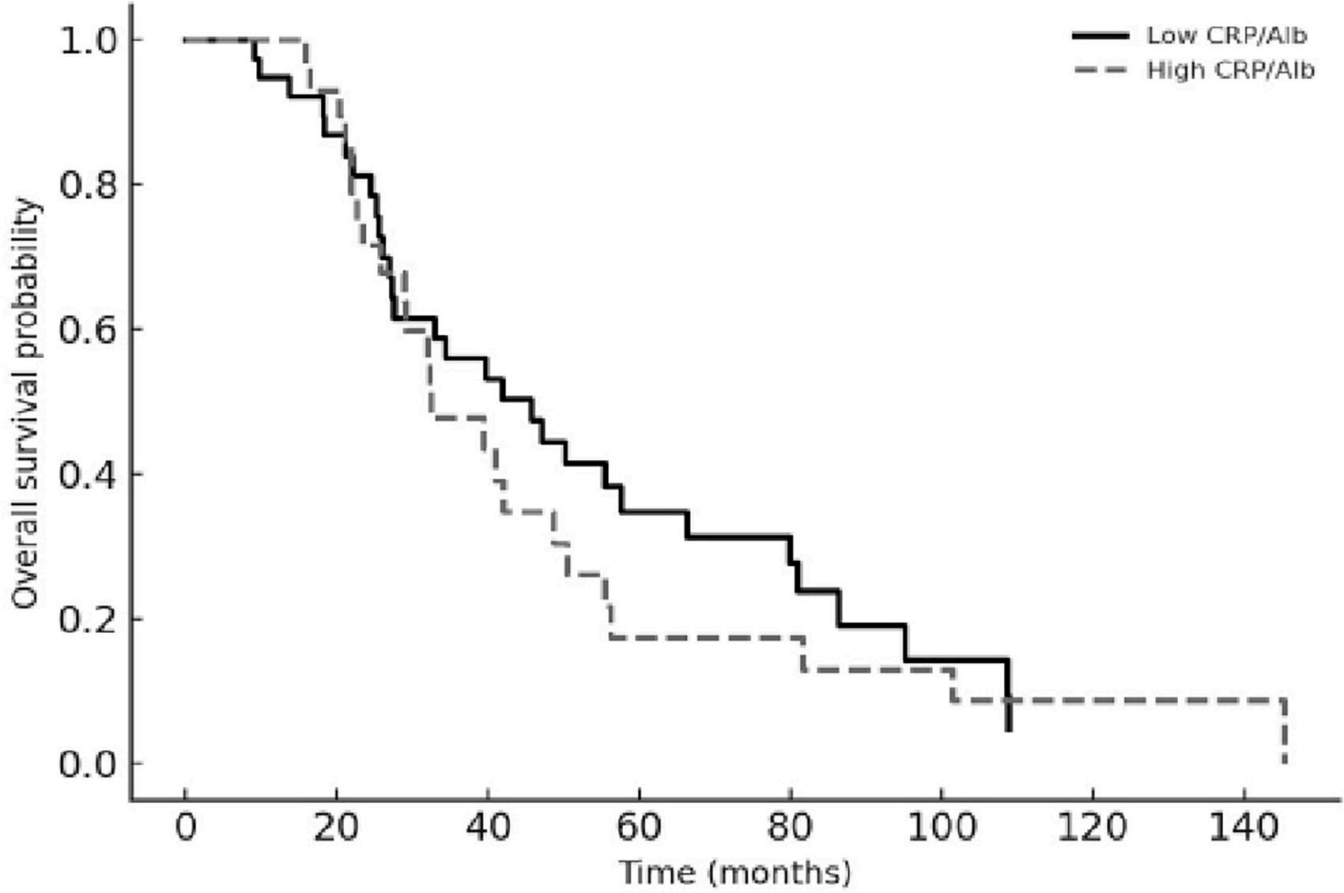
Overall survival according to baseline CRP-to-albumin ratio (CAR). Kaplan–Meier curves for overall survival stratified by baseline CAR, dichotomized at the cohort median. The solid line represents patients with low CAR and the dashed line those with high CAR. Patients with elevated CAR had significantly shorter overall survival (log-rank *p* < 0.01).

**FIGURE 3:**
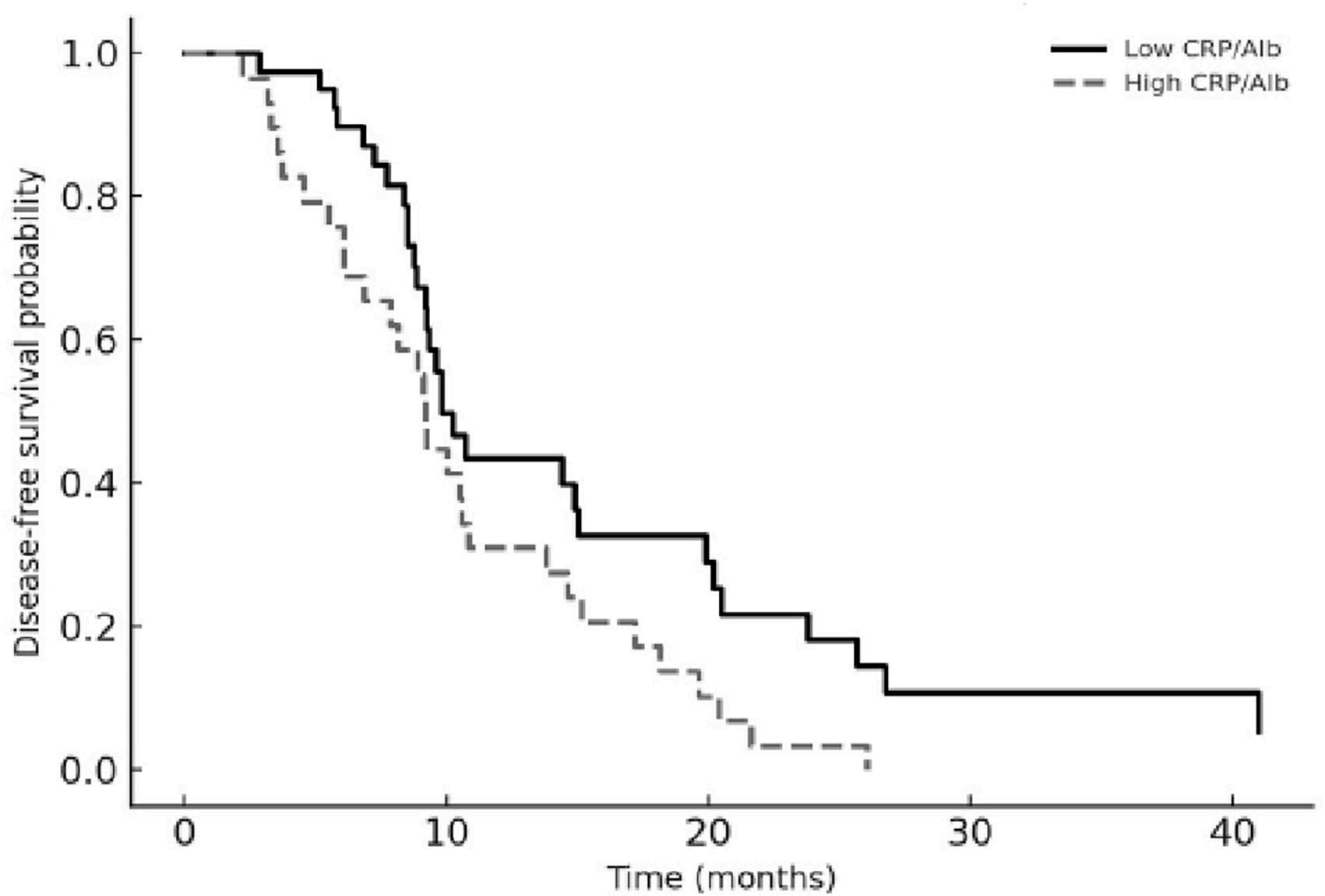
Progression-free survival according to baseline CRP-to-albumin ratio (CAR). Kaplan–Meier curves for progression-free survival stratified by baseline CAR, dichotomized at the cohort median. The solid line represents patients with low CAR and the dashed line those with high CAR. Patients with elevated CAR had significantly shorter progression-free survival (log-rank *p* < 0.01).

Kaplan–Meier analyses were consistent with these regression findings. Patients with a higher baseline CRP-to-albumin ratio had significantly shorter OS compared with those below the median, and an elevated post-TACE CRP-to-albumin ratio was associated with earlier recurrence in the PFS analysis (both log-rank *p* < 0.01). Lower dNLR values corresponded to longer survival, and patients with five or more liver lesions experienced distinctly worse PFS than those with fewer metastases. The Kaplan–Meier curves for OS and PFS according to these parameters are shown in Figures 2, 3.

ROC curve analyses were performed to assess the discriminative performance of the inflammatory and composite indices. For OS, the baseline CRP-to-albumin ratio, ΔCAR, and dNLR yielded AUC values of 0.672, 0.691, and 0.658, respectively. For PFS, ΔCAR again demonstrated the best individual performance among the single inflammatory indices (AUC 0.683).

The composite chemoembolization–tumor burden–inflammation balance score (CT-IBS) was developed by integrating ΔCAR with the number of hepatic metastases. One point was assigned for elevated ΔCAR and one point for ≥ 5 metastases, generating three risk groups: 0 (low risk), 1 (intermediate risk), and 2 (high risk). Patients in the high-risk CT-IBS category had significantly shorter OS and PFS than those in the low-risk group. Median OS values were 27.3, 17.8, and 8.6 months across the low-, intermediate-, and high-risk strata, while the corresponding PFS values were 13.4, 8.9, and 5.1 months (all log-rank *p* < 0.001).

**FIGURE 4:**
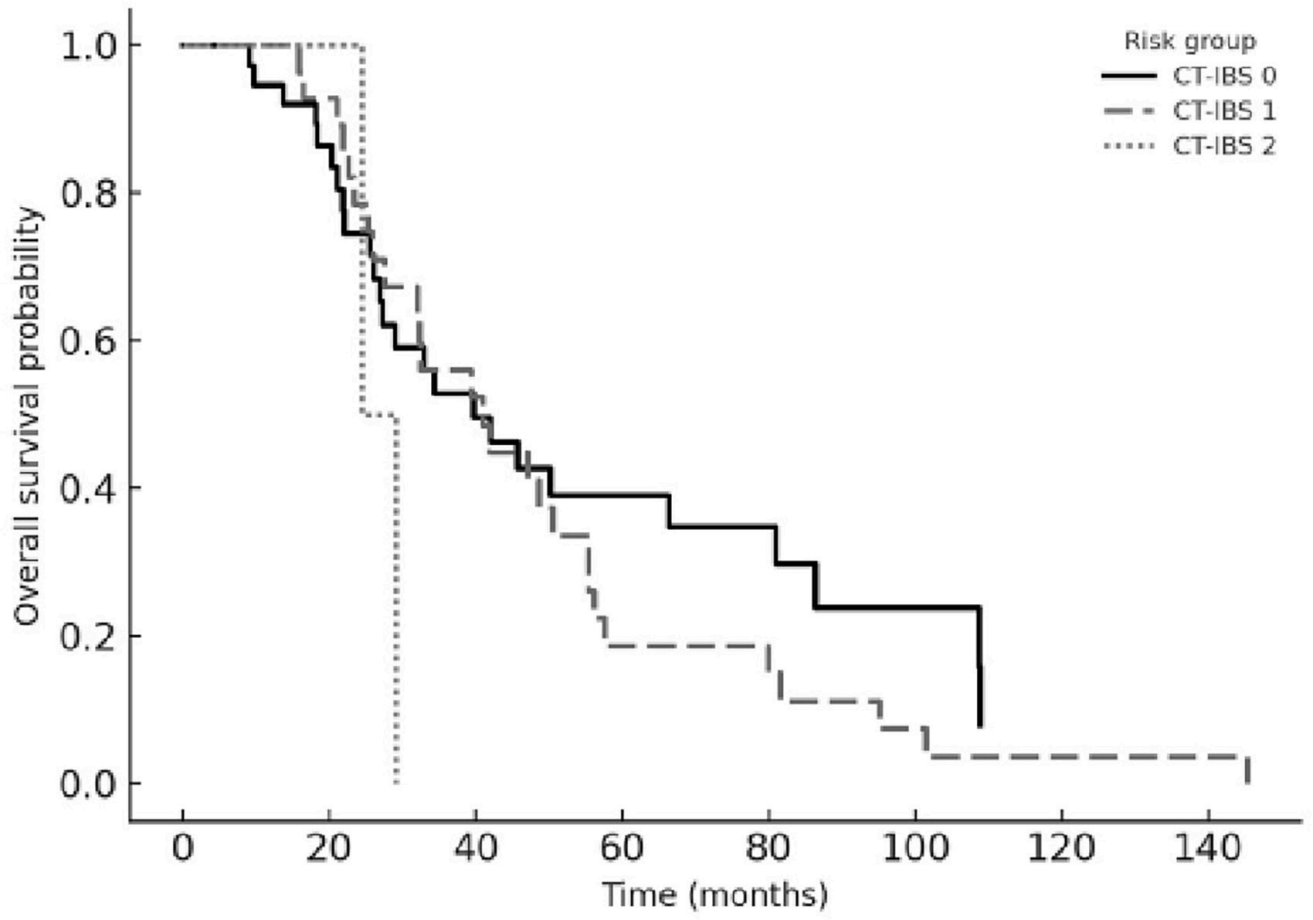
Overall survival according to CT-IBS risk groups. Kaplan–Meier curves for overall survival stratified by the chemoembolization–tumor burden– inflammation balance score (CT-IBS). CT-IBS 0 (solid line) indicates no adverse factors, CT- IBS 1 (dashed line) one adverse factor, and CT-IBS 2 (dotted line) both adverse factors (high ΔCAR and ≥ 5 liver metastases). Overall survival decreased progressively from CT-IBS 0 to CT- IBS 2 (log-rank *p* < 0.001).

**FIGURE 5:**
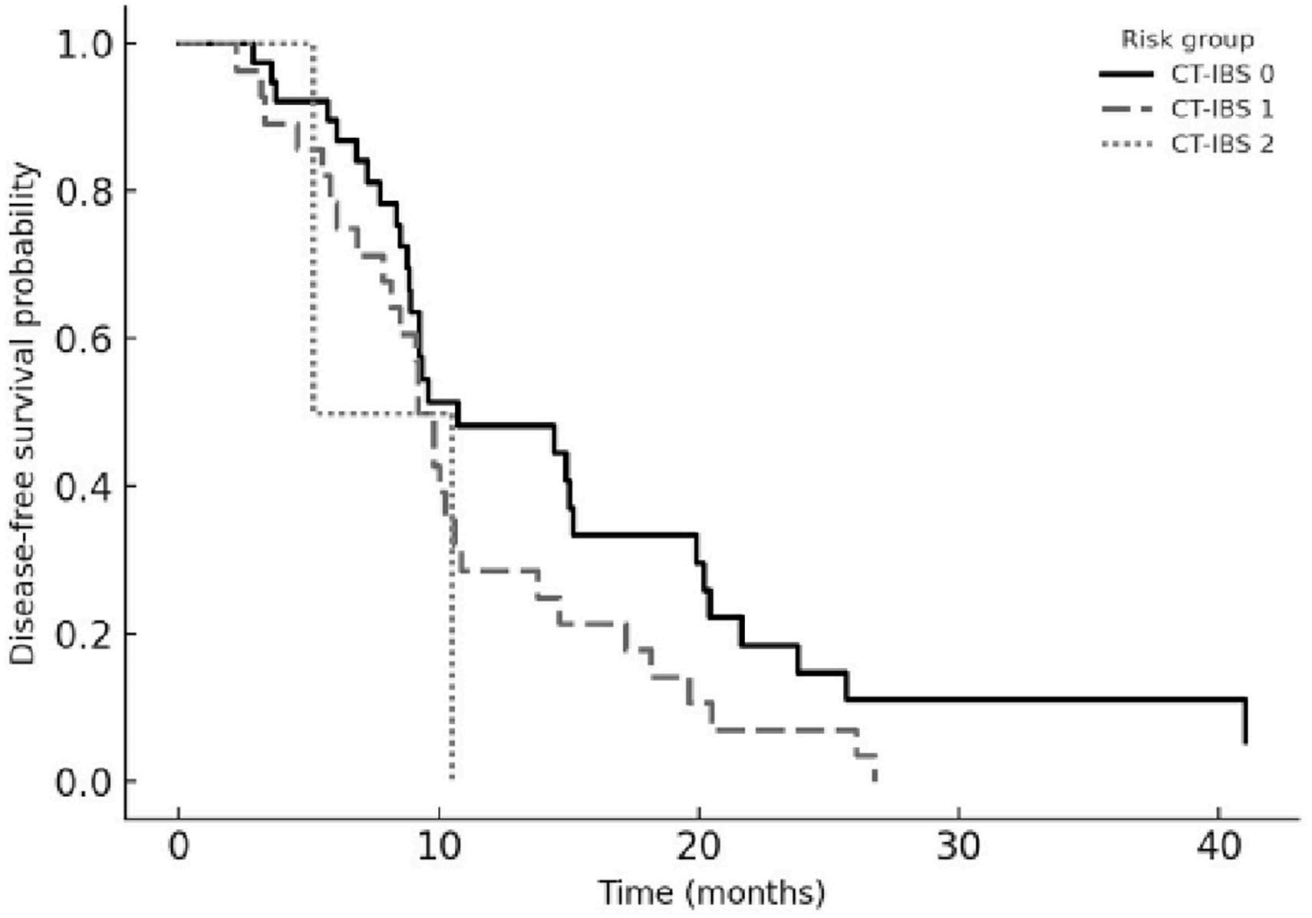
Progression-free survival according to CT-IBS risk groups. Kaplan–Meier curves for progression-free survival stratified by the chemoembolization–tumor burden–inflammation balance score (CT-IBS). CT-IBS 0 (solid line) indicates no adverse factors, CT-IBS 1 (dashed line) one adverse factor, and CT-IBS 2 (dotted line) both adverse factors (high ΔCAR and ≥ 5 liver metastases). Progression-free survival decreased progressively from CT-IBS 0 to CT-IBS 2 (log-rank *p* < 0.001).

In multivariable analyses, CT-IBS retained independent prognostic significance for both OS (HR 2.86, 95% CI 1.62–5.05; *p* < 0.001) and PFS (HR 2.43, 95% CI 1.39–4.25; *p* = 0.002). Model discrimination was acceptable, with AUC values of 0.703 for OS and 0.716 for PFS. Overall, CT-IBS outperformed individual biomarkers and created a clear risk gradient, with patients in the high-risk category having nearly a three-fold higher risk of mortality than those in the low- risk group. Kaplan–Meier curves for OS and PFS stratified by CT-IBS are shown in Figures 4, 5.

## 4 Discussion

DEBIRI-TACE is increasingly used as a liver-directed option for patients with unresectable or treatment-refractory colorectal liver metastases, but survival remains highly variable and validated prognostic tools for routine practice are still lacking (6–9). In this single-center cohort of 70 patients treated with irinotecan-loaded microsphere TACE, we observed a median OS of 18.9 months and a median PFS of 9.1 months, which compare favorably with several contemporary series reporting shorter survival under similar indications (10, 11). Building on this experience, we developed a simple composite score, the CT-IBS, which integrates dynamic systemic inflammation (ΔCAR) with hepatic tumor burden and provides clinically meaningful risk stratification after DEBIRI-TACE.

The survival outcomes observed in our cohort appear somewhat longer than those reported by Maraj et al. and Sljivic et al., who described median OS and PFS of approximately 12.7 and 5.4 months, respectively, in DEBIRI-treated populations (10, 11). These differences are likely multifactorial. Nearly all of our patients had ECOG 0–1 and liver-dominant disease, indicating that TACE was offered to relatively fit patients with preserved hepatic reserve. Most had received prior systemic therapy, which may have selected for more indolent tumor biology and better treatment tolerance. In addition, all procedures were performed within a standardized DEBIRI-TACE protocol with systematic early imaging follow-up, whereas earlier studies often included broader performance status spectra, more frequent extrahepatic disease, and heterogeneous embolization techniques (6–11). Thus, our results should not be interpreted as evidence that DEBIRI-TACE is inherently superior in this setting, but rather as an illustration of what can be achieved when patient selection and procedural execution are tightly controlled—conditions under which a structured prognostic tool such as CT-IBS may have particular utility. Baseline systemic inflammation emerged as a key determinant of outcome. Higher pretreatment CAR was independently associated with shorter OS, whereas a lower dNLR (below 3) predicted more favorable survival. These findings are consistent with the growing literature showing that inflammation-based indices capture both tumor-associated inflammatory activity and host nutritional status in colorectal cancer. Liao et al. reported that elevated CAR independently predicted worse survival across disease stages (12), while Frühling et al. and Neal et al. showed that CRP-, albumin-, and neutrophil-based scores were associated with poorer outcomes after resection of colorectal liver metastases and could outperform conventional clinicopathologic variables (13, 14). In our analysis, CAR and dNLR remained prognostic even after adjustment for established clinical factors, suggesting that systemic inflammation measured immediately before embolization conveys prognostic information that is not fully captured by tumor extent or performance status.

Dynamic changes in inflammation after TACE added further prognostic information. An increase in CAR (ΔCAR) following treatment was consistently associated with shorter OS and PFS and showed higher discriminative ability than baseline CAR in ROC analyses, although ΔCAR did not retain independent significance for OS in the fully adjusted model. This pattern mirrors observations from other settings in which dynamic inflammatory indices outperform static measurements (15–17). In the context of DEBIRI-TACE, ΔCAR probably reflects a composite signal that includes embolization-related hepatic injury, evolving tumor necrosis, and persistent tumor-driven inflammation. While our data do not allow these components to be disentangled, the consistent association between higher ΔCAR and adverse outcomes supports the use of post-procedural inflammatory change as a pragmatic marker of early biological response, rather than relying on baseline values alone.

Tumor burden also retained a central prognostic role. Patients with five or more liver metastases had significantly shorter PFS, in line with prior studies showing that extensive intrahepatic disease limits the durability of TACE even when technical success is achieved (8, 11). Our findings mirror those of Sljivic et al., who reported better outcomes in patients with fewer hepatic lesions (11), and of Vogl et al., who identified intrahepatic tumor load as a major determinant of survival across different locoregional strategies (8). Early radiologic response at 1 month further stratified prognosis, consistent with data from Chung et al. showing that morphologic response of colorectal liver metastases correlated with pathologic necrosis and survival (18). Together, these observations support the concept that both the anatomical extent of hepatic disease and the short-term imaging response remain key anchors for post-TACE risk assessment.

Against this background, CT-IBS was deliberately constructed from two variables that are routinely available and biologically complementary: ΔCAR and the number of liver metastases (≥ 5 vs. < 5). We chose these components *a priori* based on their independent associations with outcome and their non-redundant nature, rather than using data-driven selection of multiple overlapping indices. In our analysis, CT-IBS achieved higher AUC values for both OS and PFS than any single inflammatory marker and created a clear risk gradient, with high-risk patients experiencing nearly a three-fold higher mortality than low-risk patients. Similar composite models that combine inflammation-based scores with tumor burden have shown improved prognostic discrimination in hepatocellular carcinoma and resected colorectal liver metastases (19, 20), and multidimensional systemic indices such as the CLIR score have demonstrated added value in metastatic colorectal cancer (21). To our knowledge, CT-IBS is the first score to combine a dynamic inflammatory marker with hepatic tumor burden specifically to predict outcomes after DEBIRI-TACE in colorectal liver metastases. Importantly, we do not propose CT-IBS as a replacement for existing clinical judgment or staging systems, but as a simple tool that may complement them when selecting candidates for TACE, planning surveillance intervals, and identifying patients who might benefit from early systemic intensification or alternative liver-directed strategies.

We also explored the relationship between molecular alterations and TACE outcomes. Although RAS and BRAF mutations are established adverse prognostic markers and have been linked to inferior outcomes after hepatic resection or ablation (8, 22, 23), we did not observe a clear association between KRAS/BRAF/HER2 status and early radiologic response in this cohort. These neutral findings must be interpreted with caution, given the modest sample size and incomplete molecular profiling, and they do not exclude a longer-term impact of molecular status on survival. However, they suggest that, within the confines of our dataset, inflammatory and burden-based parameters captured much of the short-term risk variation that was clinically actionable, and that CT-IBS appeared to retain prognostic value irrespective of molecular profile. Systemic therapy was integrated before and after TACE in a substantial proportion of patients, reflecting real-world multimodal management. Recent studies have suggested that combining drug-eluting bead TACE with chemotherapy may improve intrahepatic disease control compared with systemic therapy alone in both first-line and pretreated settings (24, 25). Our study was not designed or powered to compare specific systemic regimens or sequences. Nevertheless, CT-IBS remained prognostic in models that included prior and post-TACE systemic therapy, supporting its potential applicability across different systemic treatment backbones and underlining that its prognostic signal is not confined to a single therapeutic context.

This study has several limitations. Its retrospective, single-center design and modest sample size limit the strength of causal inferences and reduce the precision of subgroup analyses, particularly for molecular subtypes and radiologic response categories. Systemic treatment regimens before and after TACE were heterogeneous, and some laboratory and molecular data were missing, which may have introduced residual confounding despite multivariable adjustment. Although bead size and irinotecan loading remained standardized throughout the study period, the long inclusion period may have introduced subtle variations related to operator experience and evolving clinical practice. Furthermore, a small proportion of patients underwent repeat TACE, which may introduce some treatment heterogeneity despite survival analyses being calculated from the first session. Post-TACE laboratory values were obtained at the first follow-up visit, typically coinciding with imaging assessment at approximately 4–6 weeks. This timing was not identical for all patients, and it remains uncertain whether earlier or later measurement would provide better prognostic discrimination. The optimal timing for post-procedural inflammatory assessment after DEBIRI-TACE warrants evaluation in prospective studies with standardized follow-up protocols. In addition, CT-IBS was developed and internally evaluated in the same cohort, without external validation, so some degree of optimism in its performance estimates cannot be excluded. Our findings should therefore be interpreted in the context of patients treated with DEBIRI-TACE because they were not considered candidates for curative hepatic resection at the time of treatment decision. Prospective multicenter studies are needed to validate CT-IBS in independent populations. Such studies should incorporate standardized pre- and post-TACE laboratory sampling at predefined time points and include analyses accounting for systemic treatments administered before and after TACE, as these may influence inflammatory markers and survival outcomes.

Despite these constraints, the study has notable strengths, including a well-characterized cohort treated with a standardized DEBIRI-TACE protocol, systematic early imaging follow-up, and a focus on simple, routinely available variables. By restricting CT-IBS to two non-overlapping components and limiting the complexity of the multivariable models, we aimed to reduce the risk of overfitting and to facilitate implementation in everyday practice. The consistent association of CT-IBS with both PFS and OS suggests that combining dynamic inflammation with hepatic tumor burden offers a pragmatic and biologically plausible framework for post-TACE risk stratification.

## 5 Conclusion

In patients with unresectable colorectal liver metastases treated with DEBIRI-TACE, both baseline systemic inflammation and dynamic post-procedural changes were associated with survival, and extensive hepatic tumor burden predicted earlier progression. By integrating ΔCAR with the number of liver metastases, the CT-IBS provided better prognostic discrimination than either component alone and identified a subgroup of patients at particularly high risk despite technically successful TACE. Because CT-IBS relies only on routine laboratory tests and standard imaging, it may serve as a practical adjunct to support risk-adapted follow-up and treatment planning in multidisciplinary care. Further prospective validation is required to confirm its performance in external populations and to define how best to incorporate CT-IBS into clinical decision-making algorithms for TACE-treated colorectal liver metastases.

## Data Availability

The raw data supporting the conclusions of this article will be made available by the authors, without undue reservation.
